# Endothelial Nitric Oxide Synthase Deficient Mice Are Protected from Lipopolysaccharide Induced Acute Lung Injury

**DOI:** 10.1371/journal.pone.0119918

**Published:** 2015-03-18

**Authors:** Christine M. Gross, Ruslan Rafikov, Sanjiv Kumar, Saurabh Aggarwal, P. Benson Ham III, Mary Louise Meadows, Mary Cherian-Shaw, Archana Kangath, Supriya Sridhar, Rudolf Lucas, Stephen M. Black

**Affiliations:** Pulmonary Disease Program, Vascular Biology Center, Georgia Regents University, Augusta, Georgia, United States of America; University of Illinois College of Medicine, UNITED STATES

## Abstract

Lipopolysaccharide (LPS) derived from the outer membrane of gram-negative bacteria induces acute lung injury (ALI) in mice. This injury is associated with lung edema, inflammation, diffuse alveolar damage, and severe respiratory insufficiency. We have previously reported that LPS-mediated nitric oxide synthase (NOS) uncoupling, through increases in asymmetric dimethylarginine (ADMA), plays an important role in the development of ALI through the generation of reactive oxygen and nitrogen species. Therefore, the focus of this study was to determine whether mice deficient in endothelial NOS (eNOS^-/-^) are protected against ALI. In both wild-type and eNOS^-/-^ mice, ALI was induced by the intratracheal instillation of LPS (2 mg/kg). After 24 hours, we found that eNOS^-/-^mice were protected against the LPS mediated increase in inflammatory cell infiltration, inflammatory cytokine production, and lung injury. In addition, LPS exposed eNOS^-/-^ mice had increased oxygen saturation and improved lung mechanics. The protection in eNOS^-/-^ mice was associated with an attenuated production of NO, NOS derived superoxide, and peroxynitrite. Furthermore, we found that eNOS^-/-^ mice had less RhoA activation that correlated with a reduction in RhoA nitration at Tyr^34^. Finally, we found that the reduction in NOS uncoupling in eNOS^-/-^ mice was due to a preservation of dimethylarginine dimethylaminohydrolase (DDAH) activity that prevented the LPS-mediated increase in ADMA. Together our data suggest that eNOS derived reactive species play an important role in the development of LPS-mediated lung injury.

## Introduction

Acute respiratory distress syndrome (ARDS) and acute lung injury (ALI) are severe inflammatory disorders affecting the lung. Both are characterized by non-cardiogenic pulmonary edema, hypoxemia, neutrophil infiltration, and disrupted lung mechanics [1]. The causes of ALI are diverse and can be the result of direct lung injury from viral or bacterial pneumonia, acid aspiration, and lung contusions or indirect injury as a consequence of sepsis, burns, pancreatitis, non-thoracic trauma, and multiple transfusions [2]. Lipopolysaccharide (LPS) is a component of the outer cell wall of gram-negative bacteria and is released into the body as the bacteria replicate or die [3]. LPS induces ALI in animal models by promoting pulmonary microvascular permeability and recruiting activated neutrophils and macrophages to the lung, thereby causing damage to the alveolar-capillary membrane, which leads to the deterioration of gas exchange [4]. As lung protective ventilation strategies are the only therapeutic approach that have been shown to consistently decrease mortality in ALI patients [5], there is a further need to understand the mechanisms underlying the pathology of ALI and identify new targets that can improve the outcomes of patients.

Oxidative stress has been shown to be increased in patients with ALI and is considered an important early contributor to the pathogenesis of lung injury. Our recently completed studies have shown that oxidative stress can be induced in ALI as a result of high levels of the L-arginine analogue, asymmetric dimethylarginine (ADMA) [6]. ADMA, an endogenous competitive inhibitor of the three nitric oxide (NO) synthase (NOS) isoforms, neuronal NOS (nNOS), endothelial NOS (eNOS), and inducible NOS (iNOS), displaces L-arginine from the active site [7]. In addition, ADMA can also induce the uncoupling of NOS by increasing the generation of superoxide [8] and peroxynitrite [9,10]. Peroxynitrite is a powerful nitrating agent that can affect the structure and function of proteins through the formation of 3-nitrotyrosine modifications [11]. We have shown that reducing ADMA levels is effective in both preventing, and accelerating the recovery from, LPS induced ALI [6]. In these studies, the increase in ADMA dependent NOS uncoupling and peroxynitrite generation was due to a decrease in the enzymatic activity of dimethylarginine dimethylaminohydrolase (DDAH) [6]. The two isoforms of DDAH, I and II, metabolize ADMA into L-citrulline and dimethylamine and decrease the uncoupling of NOS [12]. In addition, the increase in oxidative and nitrative stress in LPS induced ALI was linked to the nitration mediated activation of RhoA [11]. RhoA is a small GTPase and is an important regulator of the endothelial cytoskeleton and barrier function [13]. The activation of RhoA through the peroxynitrite mediated nitration at Tyr^34^ increased endothelial permeability, inflammation, and lung injury after exposure to LPS [11]. As there are reports of all three NOS isoforms being expressed in the lung [14], we utilized eNOS deficient mice to evaluate the specific role of LPS induced eNOS uncoupling on RhoA activation and lung injury in ALI. Thus, in the present study, we determined that eNOS derived peroxynitrite and protein nitration mediates the LPS induced activation of RhoA, disruption of lung mechanics, and production of pro-inflammatory cytokines. These data suggest that targeting eNOS uncoupling or RhoA activation may provide clinical benefit to patients with ALI.

## Materials and Methods

All animal breeding, housing, and protocols were approved by the Institutional Animal Care and Use Committee in facilities accredited by the American Association for the Accreditation of Laboratory Animal Care at Georgia Regents University (Augusta, GA).

### Animals and Husbandry

Breeding pairs of eNOS^-/-^ mice, strain B6.129P2-Nos3^tm1Unc^/J, stock # 002684, and wild-type (WT) mice, strain C57BL/6J, stock # 000664, were obtained from Jackson Laboratory (Bar Harbor, ME, USA) and were used to establish breeding colonies. All the animals were maintained at a room temperature of 23 ± 1°C and exposed to a 12 hour alternating light/dark cycle. The animals were fed standard rodent chow (Teklad no. 2918; Harlan Laboratories, Indianapolis, IN, USA) and given tap water *ad libitum*. The absence of eNOS expression was confirmed in all eNOS^-/-^ mice by immunoblot analysis.

### Lipopolysaccharide Induced Lung Injury Model

Adult male eNOS^-/-^ mice and wild-type mice (7–8 weeks) were used in all experiments. The mice were anesthetized with an intraperitoneal injection containing ketamine (100 mg/kg) and xylazine-HCl (10 mg/kg). The area around the throat was shaved, and the animals were placed on a heating pad. A neck midline incision was made, and the trachea was exposed. Mice then received either an intratracheal injection of *Escherichia coli* 0127:B8 LPS (2 mg/kg, Sigma-Aldrich, St. Louis, MO, USA) prepared in 0.9% saline or vehicle (0.9% saline), as previously described [11]. Mice were euthanized 24 h after LPS treatment with an intraperitoneal injection of ketamine (500 mg/kg) and xylazine-HCl (50 mg/kg), and the lungs were flushed with ice-cold EDTA-1x PBS, excised, snap-frozen in liquid nitrogen, and stored at -80°C until used.

### Generation of a Nitration-specific RhoA Polyclonal Antibody

A nitro-Tyr^34^ RhoA specific antibody was raised against a synthetic peptide antigen LLIVFSKDQFPEVY(-NO_2_)VPTVFE, where Y-NO_2_ represents 3-nitrotyrosine, as previously described [15]. The peptide was used to immunize rabbits. Tyrosine nitration-reactive rabbit antiserum was first purified by affinity chromatography. Further purification was carried out using immunodepletion using non-nitrated peptide LLIVFSKDQFPEVYVPTVFE resin chromatography, after which the resulting eluate was tested for antibody specificity by immunoblotting and immunoprecipitation followed by mass spectrometry.

### Immunoblot Analysis

Peripheral lung tissue was lysed in RIPA buffer (150 mM NaCl, 1.0% IGEPAL® CA-630, 0.5% sodium deoxycholate, 0.1% SDS, and 50 mM Tris, pH 8.0; Sigma-Aldrich) containing protease inhibitor cocktail (Sigma-Aldrich). Homogenates were then centrifuged at 20,000 g at 4°C for 20 min, the tissue supernatant was collected, and protein concentrations determined using the BCA Protein Assay (Thermo Fisher Scientific, Rockford, IL, USA). Tissue extracts (25 μg) or recombinant RhoA protein (30 μg) were resolved using 4–20% Tris-SDS-Hepes polyacrylamide gel electrophoresis, transferred to Immuno-Blot PVDF membranes (Bio-Rad Laboratories, Hercules, CA, USA), and then blocked with 5% nonfat dry milk in Tris-buffered saline containing 0.1% Tween (TBST) or 2% fish gelatin in TBST with 0.05% Tween for nitro-Tyr^34^ RhoA. The membranes were probed with antibodies against eNOS (1:1000 dilution, BD Biosciences, San Jose, CA, USA), iNOS (1:500 dilution, BD Biosciences), nNOS (1:500 dilution, custom), DDAH I (1:500 dilution, custom), DDAH II (1:500 dilution, custom), RhoA (1:500 dilution, Santa Cruz Biotechnology, Santa Cruz, CA, USA), and nitro-Tyr^34^ RhoA (1:1000 dilution in TBST with 0.05% Tween, custom). Mouse brain extract was used as a positive control for nNOS levels. Protein levels were normalized by re-probing with anti-β-actin (1:1000 dilution, Sigma-Aldrich). Reactive bands were visualized using chemiluminescence (SuperSignal West Femto substrate kit; Thermo Fisher Scientific) on a Kodak 440CF image station (New Haven, CT, USA). The band intensity was quantified using Kodak 1D image processing software, as described previously [6].

### Analysis of RhoA Activity

RhoA activity was measured using the Rhotekin Rho-binding domain pulldown assay from EMD Millipore (Billerica, MA, USA), as described previously [11]. Briefly, 30 mg of lung tissue was homogenized in Mg^2+^ Lysis Buffer (MLB), centrifuged at 20,000 g at 4°C for 20 min, the tissue supernatant was collected, and protein concentrations determined. The tissue extracts (500 μg) were then analyzed for the presence of active RhoA by precipitating GTP-bound RhoA using the Rhotekin Rho Binding Domain bound to glutathione-agarose beads. The levels of active RhoA pulled down by the assay were measured by immunoblot analysis, as described above.

### Measurement of Protein Nitration

The level of total nitrated protein was determined via a dot blot procedure, as described previously [16]. Briefly, lysates (30 μg) were applied to a nitrocellulose membrane pre-soaked with Tris-buffered saline (TBS). After the protein samples were completely transferred, the membrane was blocked in 5% nonfat dry milk in TBST for 1 h, washed with TBST, and incubated with mouse anti-3-nitrotyrosine (1:100 dilution, EMD Millipore) antibody overnight. Finally, the membrane was incubated with goat anti-mouse IgG for 2 h at room temperature. The reactive dots were visualized using chemiluminescence on a Kodak 440CF image station, as described above. The band intensity was quantified using Kodak 1D image processing software. Loading was normalized by re-probing with an anti-β-actin antibody.

### Measurement of Peroxynitrite Levels

The formation of peroxynitrite (ONOO^-^) was determined by the ONOO^-^ dependent oxidation of dihydrorhodamine (DHR) 123 to rhodamine 123, in the presence of PEG-catalase as described previously [17]. Briefly, mouse lung tissue was pulverized using a mortar and pestle; 10 mg was placed in a microfuge tube, 100 μl of 1x PBS was added, and the tissue was vortexed 3x for 10 sec. The lysate was incubated with PEG-catalase (100 U) for 30 min and was then added to a 96 well black plate in the presence of 10 μmol/L DHR123 in 1x PBS for 1 h. The fluorescence of rhodamine 123 was measured at excitation 485 nm and emission 545 nm using a Fluoroskan Ascent Fluorometer.

### Determination of NOS-Derived Superoxide

NOS-derived superoxide in mouse lung tissue was estimated using electronic paramagnetic resonance (EPR) and the spin-trap compound 1-hydroxy-3-methoxycarbonyl-2,2,5,5-tetramethylpyrrolidine HCl (CMH, Axxora LLC, Farmingdale, NY, USA), as previously described [6]. Samples were pre-incubated in the presence or absence of 100 μM ethylisothiourea (ETU, Sigma-Aldrich) for 30 min followed by incubation with CMH. All samples were analyzed for protein content using the BCA protein assay. The difference in the superoxide levels between each duplicate sample incubated in the presence or absence of ETU was used to determine NOS-dependant superoxide generation and was reported as nmols/min/mg protein.

### Measurement of NO_x_ levels

Mouse lung tissue lysates were treated with cold ethanol for 1 h at -20°C and then centrifuged at 20,000 g to remove proteins that can interfere with NO measurements, as we have previously described [18]. The potassium iodide-acetic acid reagent was prepared fresh by dissolving 0.05 g of potassium iodide in 7 ml of acetic acid. The KI/AcOH mixture was added into a septum-sealed purge vessel and bubbled with nitrogen gas. The gas stream was connected via a trap containing 1 N NaOH to a Sievers 280i Nitric Oxide Analyzer (GE Analytical, Boulder, CO, USA). The samples were injected with a syringe through a silicone-Teflon septum. The results were analyzed by measuring the area under the curve of the chemiluminescence signal using the Liquid software (GE). The resultant NO_x_ value represents total nitric oxide and nitrite in pmols per mg protein.

### Determination of ADMA Levels

ADMA levels in lung tissue homogenates were analyzed by high-performance liquid chromatography (HPLC), as previously published [6]. The crude fraction was isolated using a solid phase extraction column and subsequently, ADMA was separated using pre-column derivatization with ortho-phthaldialdehyde (OPA) reagent (4.5 mg/mL in borate buffer, pH 8.5, containing 3.3 μl/mL β-mercaptoethanol) prior to injection. HPLC was performed using a Shimadzu UFLC system with a Nucleosil phenyl reverse phase column (4.6 × 250 mm; Supelco, Bellefonte, PA, USA), equipped with an RF-10AXL fluorescence detector (Shimadzu USA Manufacturing Corporation). ADMA levels were quantified by fluorescence detection at 450 nm (emission) and 340 nm (excitation). Mobile phase A was composed of 95% potassium phosphate (50 mM, pH 6.6), 5% methanol and mobile phase B was composed of 100% methanol. ADMA was separated using a pre-gradient wash of 25% mobile phase B (flow rate 0.8 mL/min), followed by a linear increase in mobile phase B concentration from 20% to 25% over 7 min followed by a constant flow at 25% for 10 min and another linear increase from 25% to 27% mobile phase B over 5 min followed by constant flow at 27% mobile phase B for another 7 min. Retention time for ADMA was approximately 28 min. ADMA concentrations were calculated using standards and an internal homoarginine standard. The detection limit of the assay was 0.1 μmol/L.

### DDAH Activity

Total DDAH activity was determined using a radioactive assay to measure the conversion of L-[^3^H]-NMMA to [^3^H]-L-citrulline, as described previously [6]. Briefly, 20 mg of peripheral lung tissue in 125 μl of ice cold 0.1 M sodium phosphate buffer (SPB, pH 6.5) were sonicated and centrifuged at 10,000 g for 10 min at 4°C. The supernatant was collected and analyzed in duplicate (50 μl), while the remainder was used for protein concentration determination. To the supernatant, a reaction mixture was added containing 0.1 M SPB and 0.1 μCi/ml of L-[^3^H]-NMMA (specific activity: 1.48–2.96 TBq/mmol) (PerkinElmer, Santa Clara, CA, USA) in a final volume of 100 μl and incubated for 1 h at 37°C. The reaction was terminated by placing the tubes on ice for 5 min and diluting the reaction with 2 ml of ice cold SPB. The samples were then passed through 1 ml of activated Dowex AG50W-8X cation exchange resin (Sigma-Aldrich) to remove un-metabolized L-[^3^H]-NMMA followed by a rinse with 1 ml SPB. The eluted fractions were mixed with 10 ml of scintillation fluid (ScintiVerse BD Cocktail, Fisher Scientific, Pittsburgh, PA, USA) and quantified using a liquid scintillation counter. A reaction mixture containing L-[^3^H]-NMMA in the absence of enzyme was added to the Dowex column to determine background counts. DDAH activity is defined as the amount of L-[^3^H]-NMMA degraded per hour per mg protein.

### Isolation of Bronchoalveolar Lavage Fluid

Bronchoalveolar lavage fluid (BALF) was obtained by instilling and withdrawing 1 ml 1x PBS via a tracheal cannula, as described previously [6]. The cells in the BALF were pelleted at 2500 g for 10 min, and the supernatant was removed for cytokine analysis and analyzed for protein content using the BCA protein assay. The cell pellet was re-suspended in water for 15 sec to lyse the red blood cells, and then the salt concentration was normalized by the addition of 20x PBS. The total cell count of the remaining leukocytes was determined using a hemocytometer.

### Immunohistochemical Analysis of the Mouse Lung

Lungs were instilled with 10% formalin under 15 cm H_2_O pressure and immersed in the same solution before tissue processing into paraffin-embedded blocks; 4 μm sections were then cut and stained with hematoxylin and eosin (H & E). Histopathological assessment was conducted by two researchers who were masked to the treatment group. H & E stained sections were scored for the presence of leukocytes in the alveolar space, leukocytes in the interstitial space, the existence of hyaline membranes, proteinaceous debris filling the airspaces, and alveolar septal thickening, as described previously [6]. Four sections per mouse were evaluated to arrive at an average score for each animal.

### Myeloperoxidase Staining

Sections (5 μm) were cut from paraffin blocks and mounted on treated slides (Superfrost plus; Fisher Scientific). Slides were air dried overnight, placed in a 60°C oven for 30 min, deparaffinized in xylene, and run through graded alcohol to distilled water. Endogenous peroxidases were quenched with 0.3% H_2_O_2_ for 5 min followed by two rinses with distilled water. Slides were pretreated with target retrieval solution, citrate pH 6 (Dako Corporation, Carpinteria, CA, USA), rinsed in distilled water, incubated in Power Block (Biogenex Laboratories Inc, Fremont, CA, USA), rinsed in distilled water, placed in 1x PBS for 5 min then incubated with anti-myeloperoxidase (MPO) antibody (1:2000 dilution, Abcam, Cambridge, MA, USA) for 30 min at room temperature. The slides were rinsed twice in 1x PBS then incubated with a secondary peroxidase-labeled polymer conjugated to goat anti-rabbit IgG (Envision +, Dako Corp.) for 30 min, and then finally rinsed again in 1x PBS. Bound antibody was detected with diaminobenzidine (DAB+ substrate kit, Dako Corp.). Hematoxylin was used for counter-stain. MPO-stained slides were then evaluated by scoring for the presence of neutrophils within the alveolar and interstitial spaces, as described previously [6].

### Myeloperoxidase Activity

MPO activity in snap frozen mouse lung tissue was determined using a MPO Assay Kit (BioVision, Inc, Milpitas, California, USA), according to the manufacturer’s instructions, as described previously [6]. Briefly, the MPO in the samples catalyzes the production of NaClO from H_2_O_2_ and NaCl. Subsequently, the NaClO will react with exogenously added aminophenyl fluorescein to generate fluorescein, which is detected using a fluorometer using excitation at 485 nm and emission at 525 nm. The relative fluorescent units of each sample are converted into pmol of fluorescein using a standard curve. The results are reported as pmol fluorescein generated per min per mg of protein extract.

### BALF Cytokine Measurement

A panel of 32 cytokines [Eotaxin, G-CSF, GM-CSF, IFN-γ, IL-10, IL-12 (p40), IL-12 (p70), IL-13, IL-15, IL-17, IL-1α, IL-1β, IL-2, IL-3, IL-4, IL-5, IL-6, IL-7, IL-9, IP-10, KC, LIF, LIX, M-CSF, MCP-1, MIG, MIP-1α, MIP-1β, MIP-2, RANTES, TNF-α, VEGF] was assessed in duplicate in 25 μl of BALF from mice using a cytokine magnetic bead assay (MILLIPLEX MAP Mouse Cytokine/Chemokine Magnetic Bead Panel—Premixed 32 Plex—Immunology Multiplex Assay, EMD Millipore). The cytokine concentrations were measured using a MAGPIX instrument (EMD Millipore), as described previously [11].

### Assessment of Respiratory Mechanics

Twenty-four hours after LPS exposure, the mice were anesthetized with an intraperitoneal injection of ketamine (100 mg/kg) and xylazine-HCl (10 mg/kg), and the animals were placed on a heating pad. Heart rate and transcutaneous oxygen saturation were monitored via a small animal pulse oximeter (MouseOx Plus, STARR Life Sciences Corporation, Oakmont, PA, USA) by placing the non-invasive sensor on the neck, as previously described [6]. Subsequently, a neck midline incision was made to expose the trachea to facilitate endotracheal intubation with a 20-gauge 1-in.-long catheter. The animals were then connected to a FlexiVent ventilator (Scireq, Montreal, Quebec, Canada), and ventilation was initiated at 10 ml/kg tidal volume, 150/min respiratory rate, and 2.5 cm H_2_O positive end expiratory pressure (PEEP). The mice were allowed to stabilize for 5 min before measurements commenced. After two total lung capacity (TLC) maneuvers were performed (where lungs were inflated to 30 cm H_2_O), a sequence of perturbations was introduced that included a sinusoidal 1-Hz oscillation (Snapshot). The single compartment model was fit to these data using multiple linear regressions in order to calculate the dynamic elastance and compliance of the respiratory system. Dynamic pressure-volume maneuvers were also performed by stepwise increasing the airway pressure to 30 cm H_2_O and then reversing the process. After the measurement of respiratory function, the mice were disconnected from the ventilator and sacrificed by thoracotomy, as previously described [11].

### Statistical Analysis

Statistical analysis was performed using GraphPad Prism version 4.01 for Windows (GraphPad Software, San Diego, CA, USA). The mean ± SEM was calculated in all experiments, and statistical significance was determined by analysis of variance (for ≥ 3 groups). For the analysis of variance, Newman-Kuels post-hoc testing was employed. A value of p < 0.05 was considered significant.

## Results

**Fig 1:**
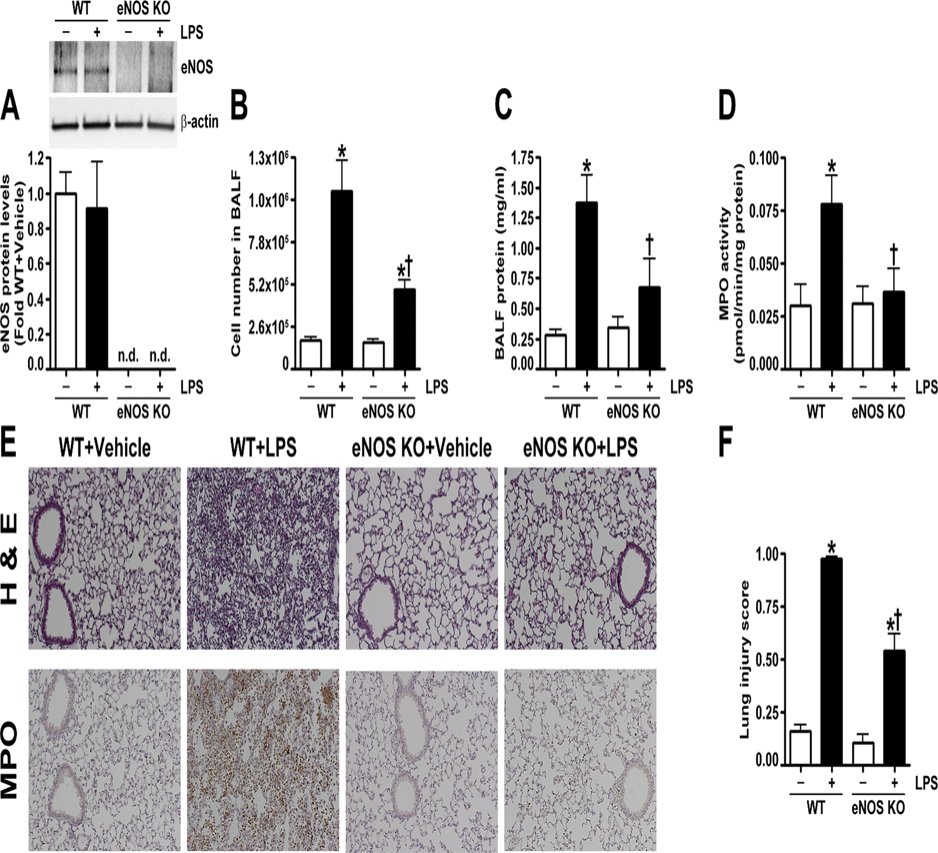
Endothelial NOS^-/-^ mice are protected from LPS mediated lung injury. Wild-type and eNOS^-/-^ mice received either saline (vehicle) or LPS (2 mg/kg body weight) intratracheally. After 24 h, the mice were anesthetized, and the lungs and bronchoalveolar lavage fluid (BALF) were collected. Protein extracts prepared from lung tissue homogenates were subjected to immunoblot analysis and probed with an anti-eNOS antibody. Densitometric analysis indicated that eNOS protein levels did not change in wild-type mice exposed to LPS and confirmed the absence of eNOS expression in eNOS^-/-^ mice (A). Total cell count in the BALF was elevated after LPS exposure in both wild-type and eNOS^-/-^ mice; although, this response was significantly decreased in the BALF of eNOS^-/-^ mice (B). Both the total protein levels in the BALF (C) and MPO activity (D) were increased in LPS treated wild-type mice but not in the BALF of LPS exposed eNOS^-/-^ mice. Lung sections were examined for signs of inflammation after hematoxylin and eosin staining (E), neutrophil infiltration after MPO staining (E), (representative micrographs are shown), and scored for lung injury (F). The inflammatory response induced by LPS in wild-type animals was reduced in eNOS^-/^- mice, as indicated by significantly less lung MPO staining (E) and a lower lung injury score (F). Values are mean ± SEM, n = 6–10. Not detected (n.d.) *p<0.05 vs. Wild-type+Vehicle, †P<0.05 vs. Wild-type+LPS.

**Fig 2:**
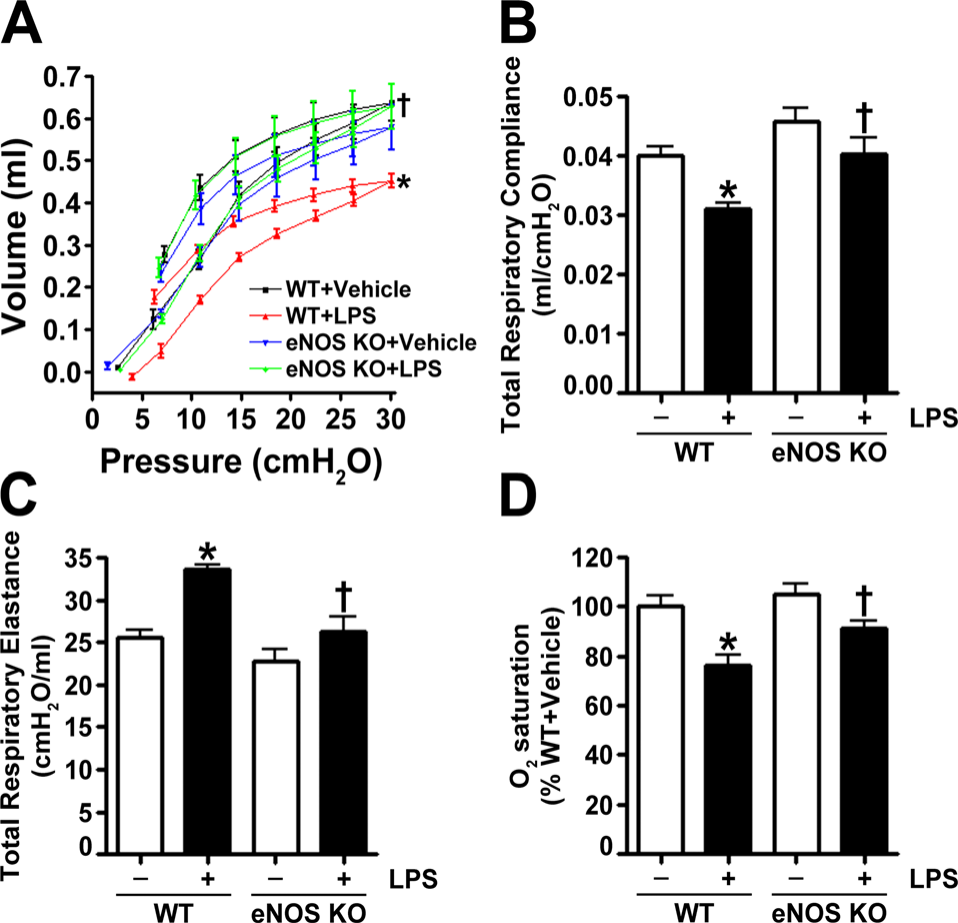
Endothelial NOS^-/-^ mice are protected from the LPS induced disruption of lung mechanics. Analysis of dynamic pressure-volume relationships in the mouse lung indicated that LPS caused a displacement of the pressure-volume curve to lower lung volumes in wild-type mice but not in eNOS^-/-^ mice (A). These data show that, for a given amount of pressure, LPS exposed wild-type mice experienced a lower tidal volume (A). The data represent pressure-volume loops for four groups with two curves: one for inhalation and one for exhalation events. Endothelial NOS^-/-^ mice exposed to LPS also had higher total respiratory compliance (B), lower total respiratory elastance (C), and increased oxygen saturation (D) compared to LPS exposed wild-type mice. Values are mean ± SEM, n = 6–10. *p<0.05 vs. Wild-type+Vehicle, †P<0.05 vs. Wild-type+LPS.

Wild-type and eNOS^-/-^ mice received either saline (vehicle) or LPS (2 mg/kg) for 24 h. We first confirmed the lack of eNOS protein in the lungs of eNOS^-/-^ mice (Fig. 1 A) and determined that eNOS protein levels did not change in wild-type mice exposed to LPS (Fig. 1A). Analysis of the BALF indicated that LPS induced cellular infiltration into the lungs after 24 h in both wild-type and eNOS^-/-^ mice (Fig. 1B). However, there was significantly less infiltration in the LPS treated eNOS^-/-^ mice (Fig. 1B). Further analysis of the BALF revealed that LPS increased protein extravasation into the airspaces of the wild-type mice but not in the eNOS^-/-^ mice (Fig. 1C). The BALF was also analyzed for the presence of 32 cytokines/chemokines. Our results demonstrate that LPS significantly increased the levels of 24 cytokines and chemokines in wild-type mice (Table 1). In eNOS^-/-^ mice, LPS increased a total of 16 cytokines, and in comparison, the LPS mediated increase in 12 cytokines was significantly lower in the eNOS^-/-^ mice than in the wild-type mice (Table 1). Using MPO activity, we found that LPS induced neutrophil infiltration in the lungs of wild-type mice but not in the lungs of eNOS^-/-^ mice (Fig. 1D). Lung sections stained with MPO and hematoxylin and eosin indicated that eNOS^-/-^ mice were protected against LPS induced histopathological changes characterized by edematous thickening of the alveolar septa, hyaline membrane formation, the infiltration of leukocytes and the presence of red blood cells in the alveolar and interstitial spaces, and debris accumulation in the alveoli (Fig. 1E). A semiquantitative histopathological scoring system [19] was also used to assess the severity of the lung injury by evaluating the extent of intra-alveolar neutrophil permeation, alveolar septal thickening, fibrin accumulation filling the airspaces, and the presence of hyaline membranes. Lung morphology was similar in both wild-type and eNOS^-/-^ vehicle treated mice; however, upon LPS stimulation, the increase in the lung injury score in the eNOS^-/-^ mice was significantly less than in the wild-type mice (Fig. 1F). An analysis of lung mechanics revealed that LPS exposure caused a downward displacement of the pressure-volume curve in wild-type mice; however, in eNOS^-/-^ mice, LPS did not affect lung mechanics (Fig. 2A). In wild-type mice exposed to LPS, lung compliance was decreased (Fig. 2B), lung elastance was increased (Fig. 2C), and oxygen saturation was reduced (Fig. 2D). In contrast, LPS instillation did not alter lung compliance (Fig. 2B), lung elastance (Fig. 2C), or oxygen saturation (Fig. 2D) in eNOS^-/-^ mice.

**Table 1:** BALF cytokines concentrations (pg/ml) are presented as Mean ±SEM for groups (WT+Vehicle: n = 6, WT+LPS: n = 10, eNOS^-/-^+Vehicle: n = 8, eNOS^-/-^+LPS: n = 9).

Cytokines	Role in lung injury	WT+Vehicle(pg/ml) n = 6	WT+LPS(pg/ml) n = 10	eNOS^-/-^+Vehicle(pg/ml) n = 8	eNOS^-/-^+LPS(pg/ml) n = 9
**IL-1 alpha**	**Pro-inflammatory interleukin**	31.76±1.95	144.38±11.26*	39.82±7.44	119.50±13.40*
**IL-1 beta**	**Pro-inflammatory interleukin**	2.34±0.77	160.87±22.84*	7.61±2.39	55.69±6.37^†^
**IL-2**	**Provides adaptive immunity**	8.19±0.80	6.58±0.26	8.15±2.33	9.89±2.23
**IL-3**	**Provides adaptive immunity**	0±0	1.46±0.41	0.51±0.51	2.34±1.17
**IL-4**	**Activates B & T cells**	0±0	0.12±0.12	0.24±0.24	1.00±0.52
**IL-5**	**Key activator of eosinophils**	1.65±0.94	15.02±2.28*	9.07±3.60	14.29±3.15
**IL-6**	**Both pro- & anti- inflammatory**	0±0	35842±7427*	64.74±50.11	4199±1553^†^
**IL-7**	**Hematopoietic growth factor**	1.77±0.22	4.44±0.27	2.58±1.37	5.64±1.90
**IL-9**	**Pro-proliferative & anti-apoptotic**	18.88±4.40	94.21±7.66*	29.26±18.72	84.80±14.67*
**IL-10**	**Anti-inflammatory**	3.16±0.62	43.45±8.53*	6.05±3.24	35.57±8.54*
**IL-12(p40)**	**Stimulate T cells, IFN-γ & TNF-α**	0.45±0.45	9.49±2.69	3.53±3.53	10.84±5.43
**IL-12(p70)**	**Stimulate T cells, IFN-γ & TNF-α**	4.89±0.98	28.00±3.61*	7.95±2.82	20.36±4.12*
**IL-13**	**Induces airway diseases**	0±0	0±0	10.51±10.51	40.37±22.25
**IL-15**	**Provides adaptive immunity**	13.43±1.24	41.92±3.33	24.06±9.38	50.11±12.65
**IL-17**	**Airway remodeling**	0±0	7.27±0.74*	0.63±0.63	8.71±1.38*
**IP-10**	**Angiogenic chemokine**	9.11±1.60	8471±2276*	64.87±37.97	1435±238.88^†^
**EOTAXIN**	**Eosinophil chemoattractant**	5.05±0.39	195.53±51.69*	15.93±4.20	55.07±13.84^†^
**G-CSF**	**Stimulates granulocyte release**	11.12±1.20	49358±2358*	853.95±745.21	55334±10850*
**GM-CSF**	**Stimulates granulocyte release**	12.53±4.17	110.67±14.33	26.26±11.59	201.19±68.62*
**IFN-gamma**	**Activator of macrophages**	1.67±0.64	49.77±21.54*	3.16±1.71	10.52±2.52^†^
**KC**	**LPS induced chemokine**	6.52±1.23	1041±250.97*	37.15±11.99	1863±662.25*
**LIF**	**Inhibits cell growth**	0±0	436.59±53.47*	27.26±23.98	168.97±68.50^†^
**LIX**	**Activates neutrophils**	0±0	352.79±62.12*	24.64±16.52	260.17±40.82*
**MCP-1**	**Recruits monocytes**	4.50±1.04	6207±2463*	66.80±46.99	2160±923.39*
**M-CSF**	**Activates macrophages**	4.12±2.00	30.44±2.51*	7.15±5.02	29.47±5.38*
**MIG**	**T cell chemoattractant**	0±0	754.27±282.59*	13.33±6.42	165.89±43.60^†^
**MIP-1 alpha**	**Recruits leukocytes**	17.96±1.56	2152±865.98*	29.03±8.90	345.90±59.38
**MIP-1 beta**	**Recruits leukocytes**	0±0	9730±269.89*	41.67±28.82	7199±1403* ^†^
**MIP-2**	**Recruits leukocytes**	106.06±1.99	4414±850.74*	120.93±20.55	2733±603.71* ^†^
**RANTES**	**Recruits leukocytes**	2.50±0.35	295.46±15.14*	4.08±1.09	199.24±33.64* ^†^
**TNF-alpha**	**Stimulates acute phase reaction**	0±0	338.67±12.55*	8.94±5.89	280.21±30.50* ^†^
**VEGF**	**Stimulates angiogenesis**	24.31±1.89	207.90±25.08*	46.57±10.07	141.78±17.90* ^†^

*p<0.05 vs. Vehicle,

^†^p<0.05 vs. WT+LPS.

Grayed rows indicate significant changes between LPS exposed wild-type mice and LPS treated eNOS^-/-^ mice. Analytes were assessed with the MCYTOMAG-70K assay. (IP-10, IFN-γ induced protein 10; G-CSF, Granulocyte colony stimulating factor; GM-CSF, Granulocyte-macrophage colony stimulating factor; KC, keratinocyte-derived chemokine; LIF, leukemia inhibitory factor; LIX, LPS-induced CXC chemokine; MCP-1, monocyte chemoattractant protein 1; M-CSF, Macrophage colony stimulating factor; MIG, monokine induced by IFN-γ; MIP, macrophage inflammatory protein; RANTES, regulated upon activation, normal T-cell expressed and secreted).

**Fig 3:**
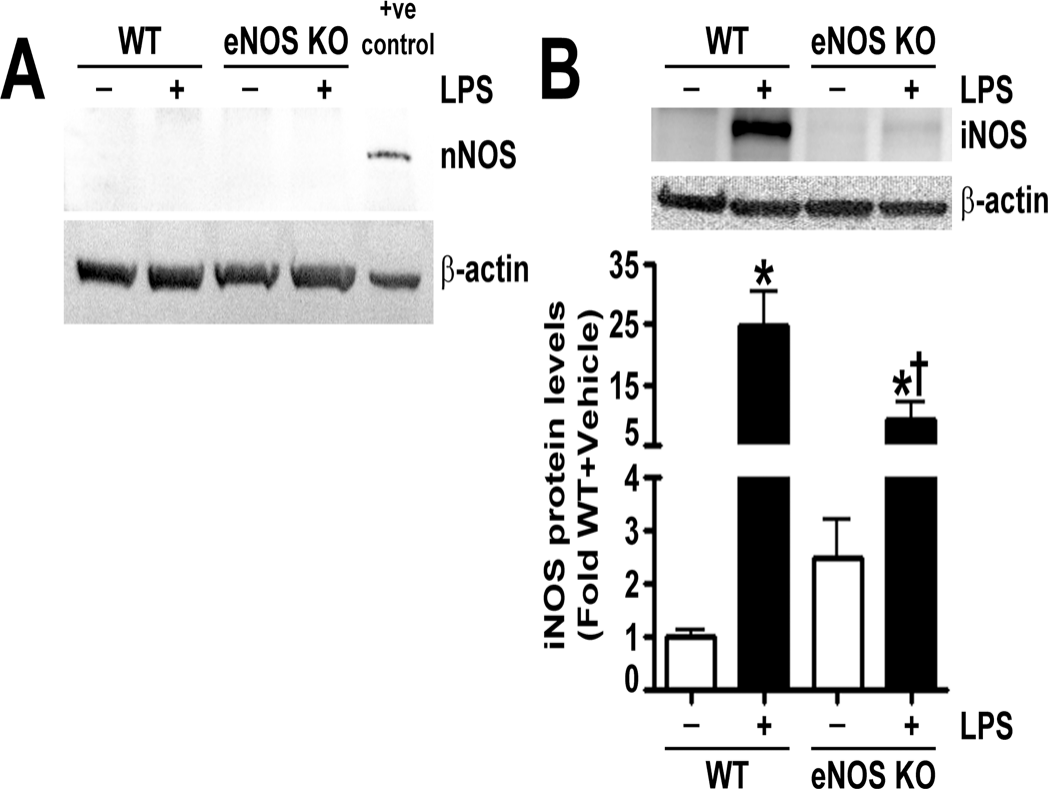
Characterization of NOS isoforms in the lungs of LPS exposed mice. Lung protein extracts were subjected to immunoblot analysis using specific antisera raised against neuronal nitric oxide synthase (nNOS) or inducible nitric oxide synthase (iNOS). Neuronal NOS was not detected in the lungs of either wild-type or eNOS-/- mice (A). The protein levels of iNOS were significantly higher in the lungs of wild-type and eNOS^-/-^ mice treated with LPS; however, the LPS induced increase in iNOS levels in the lungs of eNOS^-/-^ mice was significantly less than in the wild-type mice (B). Values are mean ± SEM, n = 6–9. *p<0.05 vs. Wild-type+Vehicle, †P<0.05 vs. Wild-type+LPS.

**Fig 4:**
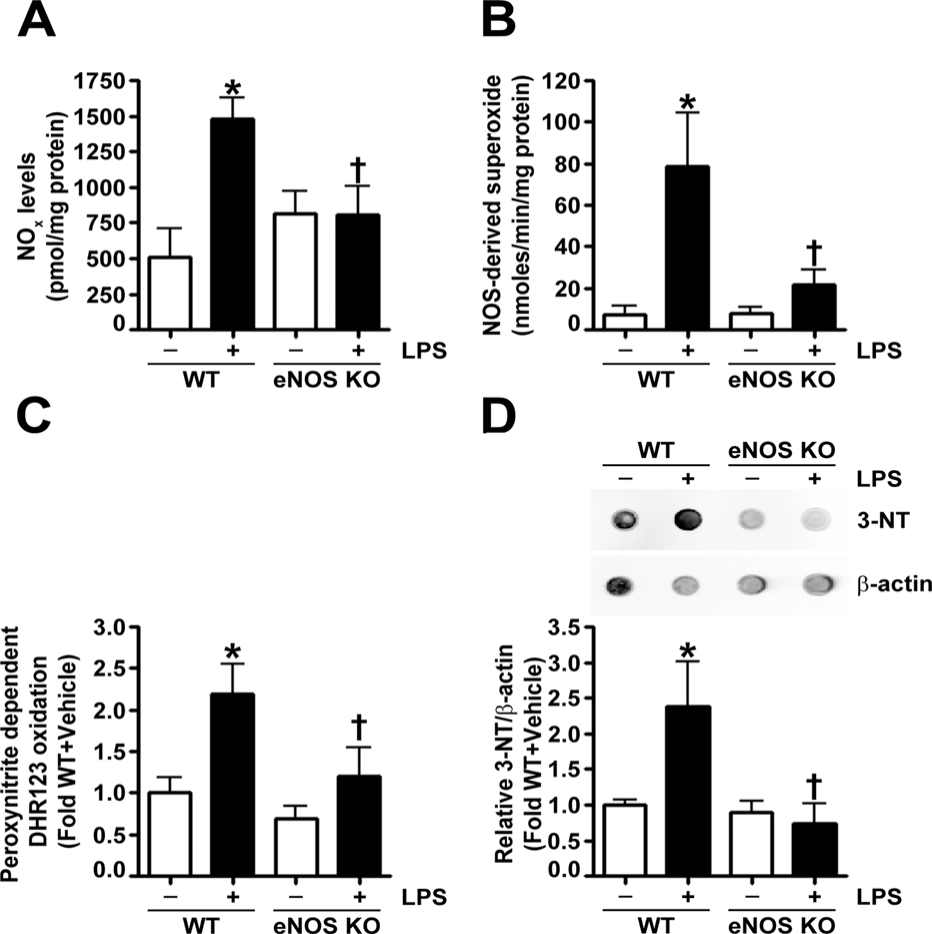
LPS dependent increases in oxidative and nitrative stress are attenuated in endothelial NOS deficient mice. The LPS mediated increase in nitric oxide (NO_x_) (A) and NOS-derived superoxide radical generation (B) was significantly lower in eNOS^-/-^ mice compared to wild-type mice. Analyses of the levels of nitrative stress, estimated using both the peroxynitrite dependent oxidation of dihydrorhodamine (DHR) 123 to rhodamine 123 (C) and 3-nitrotyrosine (3-NT) levels using dot blot analysis (D), indicate that the LPS induced increase in peroxynitrite (C) and 3-NT (D) levels in the lungs of wild-type mice was absent in LPS treated eNOS^-/-^ mice. Values are mean ± SEM, n = 6–10. *p<0.05 vs. Wild-type+Vehicle, †P<0.05 vs. Wild-type+LPS.

**Fig 5:**
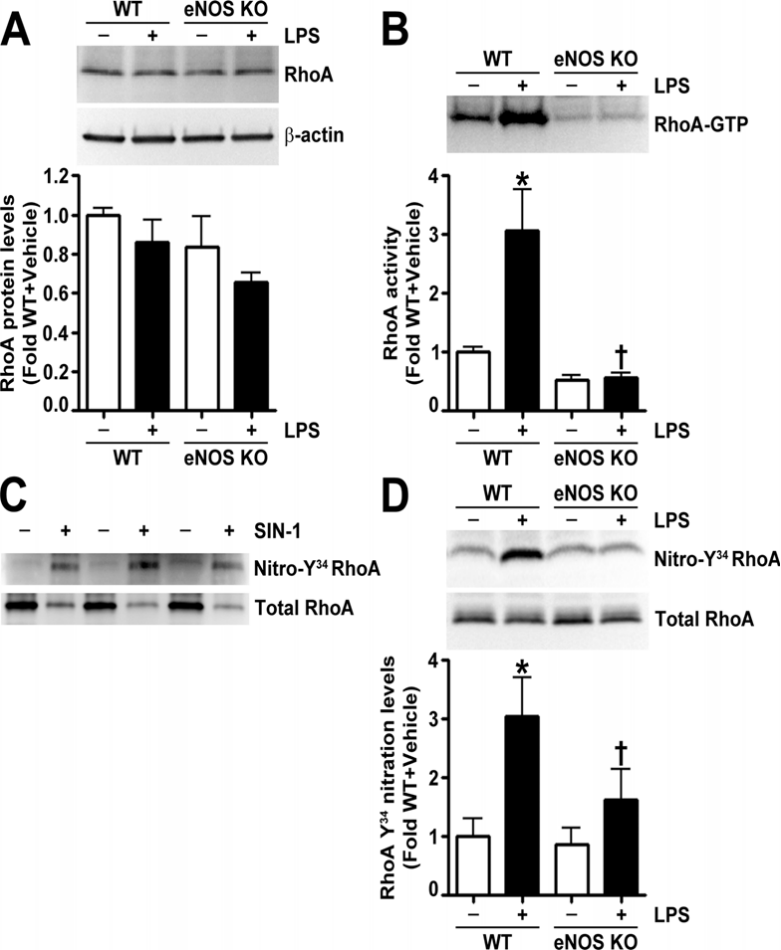
Endothelial NOS deficiency prevents LPS mediated RhoA activation and nitration at Y^34^ in the mouse lung. Immunoblot analysis of lung tissue extracts indicated no differences in RhoA protein levels in either wild-type or eNOS^-/^- mice in the absence or presence of LPS (A). However, LPS induced a significant increase in RhoA activity in the lungs of wild-type mice but not in eNOS^-/-^ mice (B). Recombinant RhoA protein (30 μg) incubated in the presence or absence of SIN-1 (25 mM, 1 h at 37°C) was immunoblotted with an antibody raised against nitro-Y^34^ RhoA and then normalized with total RhoA antibody. The nitro-Y^34^ RhoA antibody preferentially bound to nitrated RhoA (C). RhoA Y^34^ nitration levels in lung extracts were significantly elevated in LPS exposed wild-type mice; however, LPS did not alter RhoA Y^34^ nitration in the lungs of eNOS^-/-^ mice (D). Values are mean ± SEM, n = 6–10. *p<0.05 vs. Wild-type+Vehicle, †P<0.05 vs. Wild-type+LPS.

To determine the mechanism by which eNOS^-/-^ mice were protected from LPS induced lung injury, we examined the levels of the other NOS isoforms and NOS derived oxidative and nitrative stress in the lungs of LPS exposed wild-type and eNOS^-/-^ mice. We could not detect nNOS in the lungs of either wild-type or eNOS^-/-^ mice (Fig. 3A). In addition, the basal levels of iNOS were similar in both wild-type and eNOS^-/-^ mice; however, upon LPS stimulation, the increase in iNOS in the lungs of eNOS^-/-^ mice was significantly less than in the wild-type mice (Fig. 3B). Our results also show that the LPS mediated increases in NO levels (Fig. 4A), NOS-derived superoxide (Fig. 4B), peroxynitrite formation (Fig. 4C), and protein nitration (Fig. 4D) were significantly lower in the lungs of eNOS^-/-^ mice. Previously, we have shown that the LPS induced changes in mouse lung mechanics were associated with RhoA activation due to its nitration at residue Tyr^34^ [11]. We also showed that by preventing RhoA nitration and activation we could normalize lung function in LPS treated mice [11]. Twenty-four hours post-intratracheal LPS injection, we found that, although the protein levels of RhoA were unchanged (Fig. 5A), LPS induced the activation of RhoA in the lungs of wild-type mice but not in the lungs of eNOS^-/-^ mice (Fig. 5B). We then developed an antibody specific to nitro-Y^34^ RhoA to directly evaluate RhoA nitration at Tyr^34^ in mouse lung tissue. To confirm its specificity to nitrated RhoA at Tyr^34^, we subjected recombinant human RhoA protein, treated with and without the peroxynitrite donor, 5- amino- 3- (4- morpholinyl)- 1, 2, 3- oxadiazolium chloride (SIN-1), to immunoblot analysis and found that the antibody detected higher levels of nitro-Y^34^ RhoA in SIN-1 treated recombinant RhoA (Fig. 5C). Immunoblot analysis also demonstrated that, while LPS instillation increased RhoA Tyr^34^ nitration levels in wild-type mouse lungs, the nitration of RhoA at Tyr^34^ in the lungs of eNOS^-/-^ mice was not increased by LPS exposure (Fig. 5D).

**Fig 6:**
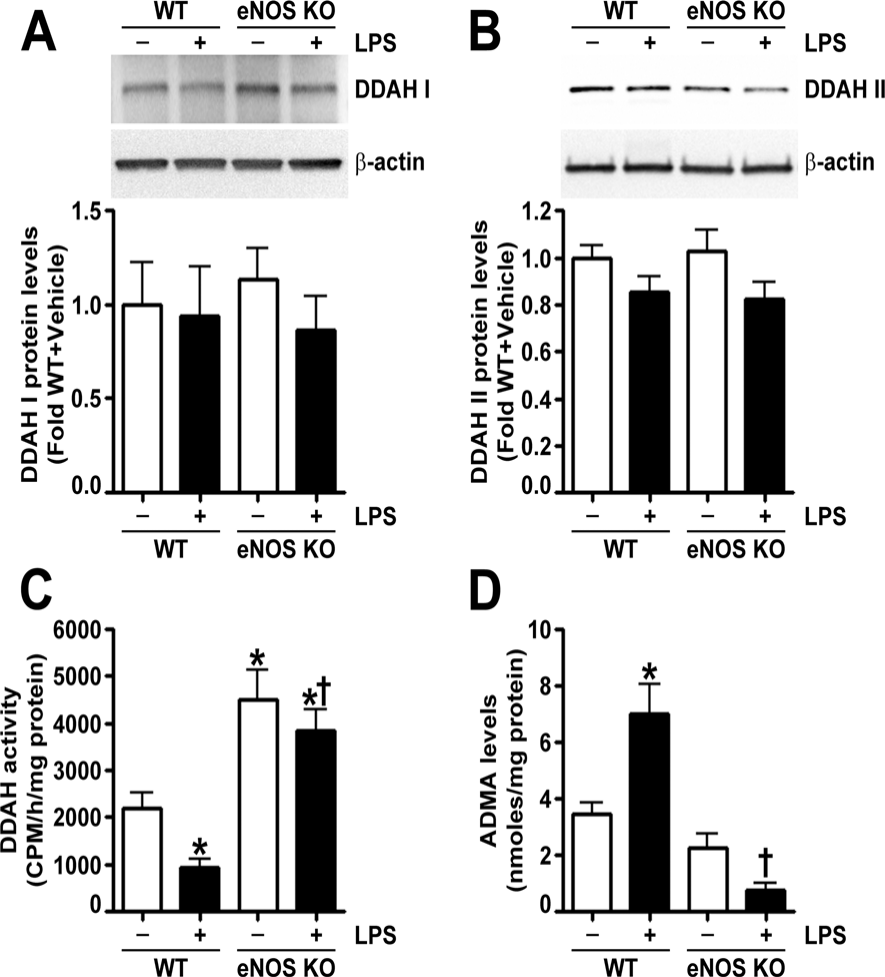
Endothelial NOS deficiency preserves DDAH activity and attenuates the LPS induced increase in ADMA in the mouse lung. Immunoblot analysis demonstrated that LPS did not change DDAH I (A) or DDAH II (B) protein levels in the lungs of either wild-type or eNOS^-/-^ mice. DDAH activity, estimated by the conversion of L-[^3^H]-NMMA to [^3^H]-L-citrulline, was significantly decreased by LPS exposure in the lungs of wild-type mice (C); however, DDAH activity was not attenuated in the lungs of LPS treated eNOS^-/-^ mice (C). In addition, eNOS^-/-^ mice exhibited increased lung DDAH activity compared to wild-type mice (C). LPS exposure increased ADMA levels in wild-type mouse lungs but not in the lungs of eNOS^-/-^ mice (D). Values are mean ± SEM, n = 6–10. *p<0.05 vs. Wild-type+Vehicle, †P<0.05 vs. Wild-type+LPS.

In a recent study, we correlated LPS induced peroxynitrite generation with increased ADMA mediated NOS uncoupling secondary to a loss of DDAH activity [6,20]. In the lungs of wild-type mice exposed to LPS, we found that, although DDAH I (Fig. 6A) and DDAH II (Fig. 6B) protein levels were not altered, total DDAH activity was decreased (Fig. 6C), and ADMA levels were increased (Fig. 6D). Similarly, in the lungs of eNOS^-/-^ mice, LPS exposure did not change DDAH I (Fig. 6A) or DDAH II (Fig. 6B) protein levels; however, DDAH activity was not attenuated (Fig. 6C), and ADMA levels were not increased (Fig. 6D). Furthermore, DDAH activity in eNOS^-/-^ mice was significantly higher than in the lungs of wild-type mice both in the presence and absence of LPS (Fig. 6C).

## Discussion

The literature investigating the involvement of individual NOS isoforms in ALI is complex with many contradictory studies. For example, although iNOS is generally considered to be the major contributor to the pathogenesis of ALI [21–29], there are opposing studies suggesting that iNOS has no effect [30,31] or is beneficial [32–34] in ALI. In addition, iNOS, as well as nNOS, has been shown to contribute to the oxidative and nitrative stress and cytokine release in sepsis induced ALI [35]. Studies have shown that the early blockade of nNOS [36] and the late inhibition of iNOS [36,37] can reduce oxidative and nitrative stress and improve outcomes in ALI [38,39]. Interestingly, our data suggest that nNOS does not play a direct role in LPS-mediated lung injury, as its expression was not detected in either wild-type or eNOS^-/-^ lung tissue, which is consistent with the findings of others [40]. The role of eNOS in the development of ALI is also controversial. Our study supports a central role for eNOS in the pathogenesis of LPS induced lung injury. Our data suggest that LPS induces eNOS uncoupling, and the subsequent increase in oxidative and nitrative stress activates RhoA resulting in endothelial barrier dysfunction and lung injury. However, a previous study found that intratracheal LPS increased lung edema, MPO activity, and the levels of pro-inflammatory cytokines, such as MIP-2, KC, MCP-1, MCP-3, to a similar extent in the BALF of wild-type and eNOS^-/-^ mice, suggesting eNOS does not play a role in the pathogenesis of ALI [32]. We similarly found that the LPS induced increases in KC and MCP-1 in the BALF were not different between wild-type and eNOS^-/-^ mice. However, we found that MPO activity and MIP-2 levels in the BALF were lower in LPS treated eNOS^-/-^ mice. The reason for these differences is unclear. In addition, studies have shown that male eNOS^-/-^ mice have elevated systemic blood pressure [41], and it is therefore possible that eNOS^-/-^ mice would be less susceptible to LPS dependent shock. Indeed, it has been shown that eNOS^-/-^ mice are resistant to LPS induced hypotension due to a reduction in iNOS protein levels [42]. Our results coincide with this study and suggest that the effects of iNOS may ultimately be attributed to eNOS, as our data show that eNOS^-/-^ mice have reduced lung iNOS expression upon exposure to LPS. This may be explained by the fact that the induction of iNOS by LPS is dependent upon the activation of NF-κB by eNOS derived NO [43,44]. However, it should be noted that eNOS deficiency may alter lung development and therefore may impact the interpretation of our results. For instance, according to previous studies, male eNOS^-/-^ mice are pulmonary hypertensive at baseline [45–47], and LPS exposure may further increase pulmonary vascular resistance and pulmonary arterial pressure. In addition, it has also been reported that neonatal lung airway development is impaired in eNOS^-/-^ mice, resulting in respiratory distress and high mortality [48]. In contrast, other studies contradict these findings and indicate that mortality is unchanged [41] and that lung development is normal in fetal, newborn [49], and adult [46,50] eNOS^-/-^ mice [41], Although, eNOS^-/-^ mice may be more susceptible to hypoxia [45,47,49,50].

It is also important to distinguish between the roles of NOS derived NO and NOS derived superoxide. Prior studies have been contradictory, demonstrating that NO is both beneficial [51–55] and harmful [56–58] in ALI. This ongoing controversy regarding the effects of NO in ALI may be attributed to the relative lack of studies assessing the specific contributions of each of the NOS isoforms, as many studies have been performed using general NOS inhibitors, which not only inhibit the activity of all the NOS isoforms but also other enzymes [59–62]. In addition, L-NAME not only inhibits NO generation from NOS but also superoxide production [63–65], while L-NMMA inhibits NO [66] but increases superoxide generation from NOS [8]. In support of this possibility it has been shown that a specific iNOS inhibitor was protective against TNF-α exposure while L-NMMA enhanced the vascular hyper-permeability response [67]. Therefore, it is important to identify the roles of each NOS isoform and to distinguish the effects of NOS derived NO and superoxide. In addition to our present study, we have previously demonstrated that LPS exposure increases NOS uncoupling and NOS derived superoxide both in vitro and in vivo [6,20]. Like the present study, the increase in NOS uncoupling was attributed to elevated ADMA levels secondary to a decrease in DDAH activity [6,20]. Interestingly, we found an increase in lung DDAH activity in eNOS^-/-^ mice, which was preserved during exposure to LPS. A previous study found that DDAH is S-nitrosylated at Cys^249^ and is reversibly inhibited by NO derived from NO donors and the cytokine induced expression of iNOS [68]. Therefore, a reduction in DDAH S-nitrosylation may explain the increase in DDAH activity and decrease in ADMA levels in LPS treated eNOS^-/-^ mice, as both iNOS expression and NO levels are attenuated. In addition, in murine lung epithelial cells, LPS and cytokine stimulation elevated the levels of ADMA which inhibited NOS and induced NOS uncoupling [10]. While the prior studies did not identify which NOS isoform is uncoupled, in our study, we found that LPS induced an increase in NOS derived superoxide in wild-type, but not in eNOS^-/-^, mice. However, as ADMA can uncouple all NOS isoforms [8,9], it is still unclear whether the reduction in NOS uncoupling in eNOS^-/-^ mice was due to the absence of eNOS or a combination of reduced iNOS protein levels, enhanced DDAH activity, and less ADMA. However, together these studies suggest that eNOS derived superoxide and peroxynitrite can contribute to lung injury in ALI.

An important mediator of NOS derived reactive species induced lung dysfunction is the small GTPase, RhoA. Recently, we have shown that the activation of RhoA through the peroxynitrite-mediated nitration at Tyr^34^ increases endothelial permeability and lung injury after exposure to LPS [11]. RhoA activation, via the stimulation of Rho kinase (ROCK), induces the formation of actin stress fibers and the destabilization of endothelial junctions by increasing myosin contractility [69]. In pulmonary endothelial cells, ADMA has been shown to increase stress fiber formation and RhoA/ROCK activation through the inhibition of NO and the subsequent attenuation of the PKG mediated Ser^188^ phosphorylation of RhoA [70] independent of ROS generation [71]. These data are partially consistent with our data, as LPS treated wild-type mice have both increases in ADMA and RhoA activation. However, eNOS^-/-^ mice exposed to LPS have reductions in both ADMA and NO, and RhoA activity is markedly decreased, suggesting that decreased Ser^188^ phosphorylation does not fully explain the mechanism of RhoA activation. RhoA/ROCK signaling can also activate NF-κB and promote inflammation in response to LPS [72] and TNF-α [73] through the canonical mechanism that involves the phosphorylation and degradation of IκBα and the translocation of NF-κB to the nucleus. Therefore, it is possible that the lower levels of several pro-inflammatory cytokines in the BALF of LPS exposed eNOS^-/-^ mice may be due to reduced lung RhoA activity. Indeed, we have previously shown that preventing RhoA nitration and reducing its activity in the lungs of LPS exposed mice reduces the levels of several cytokines in the BALF [11]. Similarly, the over-expression of DDAH II in the mouse lung prevents LPS mediated NOS uncoupling, peroxynitrite generation, and the increase in BALF inflammatory cytokines [6]. Interestingly, of the 12 cytokines that are lower in the eNOS^-/-^ mice exposed to LPS, only 5 cytokines are similarly decreased by DDAH II over-expression. As both LPS treated DDAH II over-expressing mice and eNOS^-/-^ mice have increased DDAH activity, low levels of ADMA, and decreased NOS uncoupling, these 5 cytokines: IL-6, IP-10, MIP-1β, MIP-2, and VEGF, are potentially reduced as a result of decreased NOS uncoupling and peroxynitrite generation. As we have previously shown that the nitration of IκBα at Tyr^181^ dissociates IκBα from NF-κB, and subsequently, NF-κB becomes activated [74], the decrease in oxidative and nitrative stress in LPS treated eNOS^-/-^ and DDAH II over-expressing mice may prevent the nitration of IκBα, the activation of NF-κB, reducing the expression of these inflammatory cytokines. However it is unclear why the other seven cytokines reduced in the LPS treated eNOS^-/-^ mice are not reduced in the LPS exposed DDAH II over-expressing mice and this will need further examination.

In conclusion, our data demonstrate that the oxidative and nitrative stress associated with LPS induced ALI is dependent on the presence of eNOS. Furthermore, eNOS^-/-^ mice are protected against LPS induced inflammation, lung injury, and disruption of lung mechanics. Thus, we speculate that strategies aimed at attenuating NOS uncoupling may have clinical utility. While antioxidants and other similar therapies aimed at lowering global oxidative stress have shown little efficacy in human trials, we have previously shown the benefits of using a targeted, shielding RhoA peptide in a mouse model of LPS induced ALI [11]. Further studies are warranted to determine whether preventing NOS uncoupling and RhoA nitration will provide benefit to patients with ALI.

## References

[1] Ware LB, Matthay MA (2000) The acute respiratory distress syndrome. N Engl J Med 342: 1334–1349. 10793167 10.1056/NEJM200005043421806

[2] Bernard GR, Artigas A, Brigham KL, Carlet J, Falke K, Hudson L, et al (1994) The American-European Consensus Conference on ARDS. Definitions, mechanisms, relevant outcomes, and clinical trial coordination. Am J Respir Crit Care Med 149: 818–824. 7509706 10.1164/ajrccm.149.3.7509706

[3] Lien E, Means TK, Heine H, Yoshimura A, Kusumoto S, Fukase K, et al (2000) Toll-like receptor 4 imparts ligand-specific recognition of bacterial lipopolysaccharide. J Clin Invest 105: 497–504. 10683379 10.1172/JCI8541 PMC289161

[4] Kabir K, Gelinas JP, Chen M, Chen D, Zhang D, Luo X, et al (2002) Characterization of a murine model of endotoxin-induced acute lung injury. Shock 17: 300–303. 11954830 10.1097/00024382-200204000-00010

[5] Matthay MA, Ware LB, Zimmerman GA (2012) The acute respiratory distress syndrome. J Clin Invest 122: 2731–2740. 10.1172/JCI60331 22850883 PMC3408735

[6] Aggarwal S, Gross CM, Kumar S, Dimitropoulou C, Sharma S, Gorshkov BA, et al (2014) Dimethylarginine dimethylaminohydrolase II overexpression attenuates LPS-mediated lung leak in acute lung injury. Am J Respir Cell Mol Biol 50: 614–625. 10.1165/rcmb.2013-0193OC 24134589 PMC4068933

[7] Vallance P, Leone A, Calver A, Collier J, Moncada S (1992) Accumulation of an endogenous inhibitor of nitric oxide synthesis in chronic renal failure. Lancet 339: 572–575. 1347093 10.1016/0140-6736(92)90865-z

[8] Druhan LJ, Forbes SP, Pope AJ, Chen CA, Zweier JL, Cardounel AJ (2008) Regulation of eNOS-derived superoxide by endogenous methylarginines. Biochemistry 47: 7256–7263. 10.1021/bi702377a 18553936

[9] Sud N, Wells SM, Sharma S, Wiseman DA, Wilham J, Black SM (2008) Asymmetric dimethylarginine inhibits HSP90 activity in pulmonary arterial endothelial cells: role of mitochondrial dysfunction. Am J Physiol Cell Physiol 294: C1407–1418. 10.1152/ajpcell.00384.2007 18385287 PMC3815615

[10] Wells SM, Holian A (2007) Asymmetric dimethylarginine induces oxidative and nitrosative stress in murine lung epithelial cells. Am J Respir Cell Mol Biol 36: 520–528. 17158357 10.1165/rcmb.2006-0302SM PMC1899333

[11] Rafikov R, Dimitropoulou C, Aggarwal S, Kangath A, Gross C, Pardo D, et al (2014) Lipopolysaccharide-induced lung injury involves the nitration-mediated activation of RhoA. J Biol Chem 289: 4710–4722. 10.1074/jbc.M114.547596 24398689 PMC3931033

[12] Boger RH (2004) Asymmetric dimethylarginine, an endogenous inhibitor of nitric oxide synthase, explains the "L-arginine paradox" and acts as a novel cardiovascular risk factor. J Nutr 134: 2842S–2847S; discussion 2853S. 15465797 10.1093/jn/134.10.2842S

[13] Spindler V, Schlegel N, Waschke J (2010) Role of GTPases in control of microvascular permeability. Cardiovasc Res 87: 243–253. 10.1093/cvr/cvq086 20299335

[14] Fagan KA, Tyler RC, Sato K, Fouty BW, Morris KG Jr., Huang PL, et al (1999) Relative contributions of endothelial, inducible, and neuronal NOS to tone in the murine pulmonary circulation. Am J Physiol 277: L472–478. 10484454 10.1152/ajplung.1999.277.3.L472

[15] Aggarwal S, Gross CM, Rafikov R, Kumar S, Fineman JR, Ludewig B, et al (2014) Nitration of tyrosine 247 inhibits protein kinase G-1alpha activity by attenuating cyclic guanosine monophosphate binding. J Biol Chem 289: 7948–7961. 10.1074/jbc.M113.534313 24469460 PMC3953305

[16] Aggarwal S, Dimitropoulou C, Lu Q, Black SM, Sharma S (2012) Glutathione supplementation attenuates lipopolysaccharide-induced mitochondrial dysfunction and apoptosis in a mouse model of acute lung injury. Front Physiol 3: 161 10.3389/fphys.2012.00161 22654772 PMC3361071

[17] Aggarwal S, Gross CM, Kumar S, Datar S, Oishi P, Kalkan G, et al (2011) Attenuated vasodilatation in lambs with endogenous and exogenous activation of cGMP signaling: role of protein kinase G nitration. J Cell Physiol 226: 3104–3113. 10.1002/jcp.22692 21351102 PMC3527072

[18] Sharma S, Aramburo A, Rafikov R, Sun X, Kumar S, Oishi PE, et al (2013) L-carnitine preserves endothelial function in a lamb model of increased pulmonary blood flow. Pediatr Res 74: 39–47. 10.1038/pr.2013.71 23628882 PMC3709010

[19] Matute-Bello G, Winn RK, Jonas M, Chi EY, Martin TR, Liles WC (2001) Fas (CD95) induces alveolar epithelial cell apoptosis in vivo: implications for acute pulmonary inflammation. Am J Pathol 158: 153–161. 11141488 10.1016/S0002-9440(10)63953-3 PMC1850249

[20] Sharma S, Smith A, Kumar S, Aggarwal S, Rehmani I, Snead C, et al (2010) Mechanisms of nitric oxide synthase uncoupling in endotoxin-induced acute lung injury: role of asymmetric dimethylarginine. Vascul Pharmacol 52: 182–190. 10.1016/j.vph.2009.11.010 19962451 PMC2879579

[21] Arkovitz MS, Wispe JR, Garcia VF, Szabo C (1996) Selective inhibition of the inducible isoform of nitric oxide synthase prevents pulmonary transvascular flux during acute endotoxemia. J Pediatr Surg 31: 1009–1015. 8863222 10.1016/s0022-3468(96)90075-5

[22] Wei XQ, Charles IG, Smith A, Ure J, Feng GJ, Huang FP, et al (1995) Altered immune responses in mice lacking inducible nitric oxide synthase. Nature 375: 408–411. 7539113 10.1038/375408a0

[23] Kristof AS, Goldberg P, Laubach V, Hussain SN (1998) Role of inducible nitric oxide synthase in endotoxin-induced acute lung injury. Am J Respir Crit Care Med 158: 1883–1889. 9847282 10.1164/ajrccm.158.6.9802100

[24] Vromen A, Arkovitz MS, Zingarelli B, Salzman AL, Garcia VF, Szabo C (1996) Low-level expression and limited role for the inducible isoform of nitric oxide synthase in the vascular hyporeactivity and mortality associated with cecal ligation and puncture in the rat. Shock 6: 248–253. 8902940 10.1097/00024382-199610000-00004

[25] Shelton JL, Wang L, Cepinskas G, Sandig M, Scott JA, North ML, et al (2007) Inducible NO synthase (iNOS) in human neutrophils but not pulmonary microvascular endothelial cells (PMVEC) mediates septic protein leak in vitro. Microvasc Res 74: 23–31. 17451752 10.1016/j.mvr.2007.02.008

[26] Ruetten H, Southan GJ, Abate A, Thiemermann C (1996) Attenuation of endotoxin-induced multiple organ dysfunction by 1-amino-2-hydroxy-guanidine, a potent inhibitor of inducible nitric oxide synthase. Br J Pharmacol 118: 261–270. 8735625 10.1111/j.1476-5381.1996.tb15397.x PMC1909642

[27] Kengatharan KM, De Kimpe SJ, Thiemermann C (1996) Role of nitric oxide in the circulatory failure and organ injury in a rodent model of gram-positive shock. Br J Pharmacol 119: 1411–1421. 8968550 10.1111/j.1476-5381.1996.tb16053.x PMC1915817

[28] De Kimpe SJ, Hunter ML, Bryant CE, Thiemermann C, Vane JR (1995) Delayed circulatory failure due to the induction of nitric oxide synthase by lipoteichoic acid from Staphylococcus aureus in anaesthetized rats. Br J Pharmacol 114: 1317–1323. 7542534 10.1111/j.1476-5381.1995.tb13349.x PMC1510350

[29] Koizumi T, Ogasawara H, Yamamato H, Tsushima K, Ruan Z, Jian M, et al (2004) Effect of ONO1714, a specific inducible nitric oxide synthase inhibitor, on lung lymph filtration and gas exchange during endotoxemia in unanesthetized sheep. Anesthesiology 101: 59–65. 15220772 10.1097/00000542-200407000-00011

[30] Laubach VE, Shesely EG, Smithies O, Sherman PA (1995) Mice lacking inducible nitric oxide synthase are not resistant to lipopolysaccharide-induced death. Proc Natl Acad Sci U S A 92: 10688–10692. 7479866 10.1073/pnas.92.23.10688 PMC40677

[31] MacMicking JD, Nathan C, Hom G, Chartrain N, Fletcher DS, Trumbauer M, et al (1995) Altered responses to bacterial infection and endotoxic shock in mice lacking inducible nitric oxide synthase. Cell 81: 641–650. 7538909 10.1016/0092-8674(95)90085-3

[32] Speyer CL, Neff TA, Warner RL, Guo RF, Sarma JV, Riedemann NC, et al (2003) Regulatory effects of iNOS on acute lung inflammatory responses in mice. Am J Pathol 163: 2319–2328. 14633605 10.1016/S0002-9440(10)63588-2 PMC1892362

[33] Hickey MJ, Sharkey KA, Sihota EG, Reinhardt PH, Macmicking JD, Nathan C, et al (1997) Inducible nitric oxide synthase-deficient mice have enhanced leukocyte-endothelium interactions in endotoxemia. FASEB J 11: 955–964. 9337148 10.1096/fasebj.11.12.9337148

[34] Zeidler PC, Millecchia LM, Castranova V (2004) Role of inducible nitric oxide synthase-derived nitric oxide in lipopolysaccharide plus interferon-gamma-induced pulmonary inflammation. Toxicol Appl Pharmacol 195: 45–54. 14962504 10.1016/j.taap.2003.10.005

[35] Lange M, Nakano Y, Traber DL, Hamahata A, Esechie A, Jonkam C, et al (2010) Role of different nitric oxide synthase isoforms in a murine model of acute lung injury and sepsis. Biochem Biophys Res Commun 399: 286–291. 10.1016/j.bbrc.2010.07.071 20655878

[36] Lange M, Hamahata A, Traber DL, Nakano Y, Traber LD, Enkhbaatar P (2011) Specific inhibition of nitric oxide synthases at different time points in a murine model of pulmonary sepsis. Biochem Biophys Res Commun 404: 877–881. 10.1016/j.bbrc.2010.12.078 21184738

[37] Okamoto I, Abe M, Shibata K, Shimizu N, Sakata N, Katsuragi T, et al (2000) Evaluating the role of inducible nitric oxide synthase using a novel and selective inducible nitric oxide synthase inhibitor in septic lung injury produced by cecal ligation and puncture. Am J Respir Crit Care Med 162: 716–722. 10934111 10.1164/ajrccm.162.2.9907039

[38] Lange M, Connelly R, Traber DL, Hamahata A, Cox RA, Nakano Y, et al (2009) Combined neuronal and inducible nitric oxide synthase inhibition in ovine acute lung injury. Crit Care Med 37: 223–229. 10.1097/CCM.0b013e3181926104 19050630 PMC3779893

[39] Lange M, Hamahata A, Traber DL, Nakano Y, Esechie A, Jonkam C, et al (2010) Effects of early neuronal and delayed inducible nitric oxide synthase blockade on cardiovascular, renal, and hepatic function in ovine sepsis. Anesthesiology 113: 1376–1384. 10.1097/ALN.0b013e3181fc5588 21068663

[40] Feletou M, Lonchampt M, Coge F, Galizzi JP, Bassoullet C, Merial C, et al (2001) Regulation of murine airway responsiveness by endothelial nitric oxide synthase. Am J Physiol Lung Cell Mol Physiol 281: L258–267. 11404269 10.1152/ajplung.2001.281.1.L258

[41] Miller AA, Hislop AA, Vallance PJ, Haworth SG (2005) Deletion of the eNOS gene has a greater impact on the pulmonary circulation of male than female mice. Am J Physiol Lung Cell Mol Physiol 289: L299–306. 15821017 10.1152/ajplung.00022.2005

[42] Connelly L, Madhani M, Hobbs AJ (2005) Resistance to endotoxic shock in endothelial nitric-oxide synthase (eNOS) knock-out mice: a pro-inflammatory role for eNOS-derived no in vivo. J Biol Chem 280: 10040–10046. 15647265 10.1074/jbc.M411991200

[43] Connelly L, Jacobs AT, Palacios-Callender M, Moncada S, Hobbs AJ (2003) Macrophage endothelial nitric-oxide synthase autoregulates cellular activation and pro-inflammatory protein expression. J Biol Chem 278: 26480–26487. 12740377 10.1074/jbc.M302238200

[44] Vo PA, Lad B, Tomlinson JA, Francis S, Ahluwalia A (2005) autoregulatory role of endothelium-derived nitric oxide (NO) on Lipopolysaccharide-induced vascular inducible NO synthase expression and function. J Biol Chem 280: 7236–7243. 15583003 10.1074/jbc.M411317200

[45] Fagan KA, Fouty BW, Tyler RC, Morris KG Jr., Hepler LK, Sato K, et al (1999) The pulmonary circulation of homozygous or heterozygous eNOS-null mice is hyperresponsive to mild hypoxia. J Clin Invest 103: 291–299. 9916141 10.1172/JCI3862 PMC407877

[46] Steudel W, Ichinose F, Huang PL, Hurford WE, Jones RC, Bevan JA, et al (1997) Pulmonary vasoconstriction and hypertension in mice with targeted disruption of the endothelial nitric oxide synthase (NOS 3) gene. Circ Res 81: 34–41. 9201025 10.1161/01.res.81.1.34

[47] Steudel W, Scherrer-Crosbie M, Bloch KD, Weimann J, Huang PL, Jones RC, et al (1998) Sustained pulmonary hypertension and right ventricular hypertrophy after chronic hypoxia in mice with congenital deficiency of nitric oxide synthase 3. J Clin Invest 101: 2468–2477. 9616218 10.1172/JCI2356 PMC508836

[48] Han RN, Babaei S, Robb M, Lee T, Ridsdale R, Ackerley C, et al (2004) Defective lung vascular development and fatal respiratory distress in endothelial NO synthase-deficient mice: a model of alveolar capillary dysplasia? Circ Res 94: 1115–1123. 15016731 10.1161/01.RES.0000125624.85852.1E

[49] Balasubramaniam V, Tang JR, Maxey A, Plopper CG, Abman SH (2003) Mild hypoxia impairs alveolarization in the endothelial nitric oxide synthase-deficient mouse. Am J Physiol Lung Cell Mol Physiol 284: L964–971. 12588707 10.1152/ajplung.00421.2002

[50] Balasubramaniam V, Maxey AM, Morgan DB, Markham NE, Abman SH (2006) Inhaled NO restores lung structure in eNOS-deficient mice recovering from neonatal hypoxia. Am J Physiol Lung Cell Mol Physiol 291: L119–127. 16443642 10.1152/ajplung.00395.2005

[51] Pheng LH, Francoeur C, Denis M (1995) The involvement of nitric oxide in a mouse model of adult respiratory distress syndrome. Inflammation 19: 599–610. 8543374 10.1007/BF01539139

[52] Iuvone T, D'Acquisto F, Carnuccio R, Di Rosa M (1996) Nitric oxide inhibits LPS-induced tumor necrosis factor synthesis in vitro and in vivo. Life Sci 59: PL207–211. 8809229 10.1016/0024-3205(96)00425-0

[53] Bloomfield GL, Holloway S, Ridings PC, Fisher BJ, Blocher CR, Sholley M, et al (1997) Pretreatment with inhaled nitric oxide inhibits neutrophil migration and oxidative activity resulting in attenuated sepsis-induced acute lung injury. Crit Care Med 25: 584–593. 9142021 10.1097/00003246-199704000-00006

[54] Peng HB, Libby P, Liao JK (1995) Induction and stabilization of I kappa B alpha by nitric oxide mediates inhibition of NF-kappa B. J Biol Chem 270: 14214–14219. 7775482 10.1074/jbc.270.23.14214

[55] Kubes P, Suzuki M, Granger DN (1991) Nitric oxide: an endogenous modulator of leukocyte adhesion. Proc Natl Acad Sci U S A 88: 4651–4655. 1675786 10.1073/pnas.88.11.4651 PMC51723

[56] Ader F, Le Berre R, Lancel S, Faure K, Viget NB, Nowak E, et al (2007) Inhaled nitric oxide increases endothelial permeability in Pseudomonas aeruginosa pneumonia. Intensive Care Med 33: 503–510. 17219196 10.1007/s00134-006-0497-7

[57] Wu CH, Chen TL, Chen TG, Ho WP, Chiu WT, Chen RM (2003) Nitric oxide modulates pro- and anti-inflammatory cytokines in lipopolysaccharide-activated macrophages. J Trauma 55: 540–545. 14501900 10.1097/01.TA.0000033496.62796.3B

[58] Laszlo F, Whittle BJ, Evans SM, Moncada S (1995) Association of microvascular leakage with induction of nitric oxide synthase: effects of nitric oxide synthase inhibitors in various organs. Eur J Pharmacol 283: 47–53. 7498320 10.1016/0014-2999(95)00281-o

[59] Peterson DA, Peterson DC, Archer S, Weir EK (1992) The non specificity of specific nitric oxide synthase inhibitors. Biochem Biophys Res Commun 187: 797–801. 1382421 10.1016/0006-291x(92)91266-s

[60] Buxton IL, Cheek DJ, Eckman D, Westfall DP, Sanders KM, Keef KD (1993) NG-nitro L-arginine methyl ester and other alkyl esters of arginine are muscarinic receptor antagonists. Circ Res 72: 387–395. 7678206 10.1161/01.res.72.2.387

[61] Suda O, Tsutsui M, Morishita T, Tanimoto A, Horiuchi M, Tasaki H, et al (2002) Long-term treatment with N(omega)-nitro-L-arginine methyl ester causes arteriosclerotic coronary lesions in endothelial nitric oxide synthase-deficient mice. Circulation 106: 1729–1735. 12270870 10.1161/01.cir.0000029749.16101.44

[62] Suda O, Tsutsui M, Morishita T, Tasaki H, Ueno S, Nakata S, et al (2004) Asymmetric dimethylarginine produces vascular lesions in endothelial nitric oxide synthase-deficient mice: involvement of renin-angiotensin system and oxidative stress. Arterioscler Thromb Vasc Biol 24: 1682–1688. 15217805 10.1161/01.ATV.0000136656.26019.6e

[63] Xia Y, Tsai AL, Berka V, Zweier JL (1998) Superoxide generation from endothelial nitric-oxide synthase. A Ca2+/calmodulin-dependent and tetrahydrobiopterin regulatory process. J Biol Chem 273: 25804–25808. 9748253 10.1074/jbc.273.40.25804

[64] Xia Y, Dawson VL, Dawson TM, Snyder SH, Zweier JL (1996) Nitric oxide synthase generates superoxide and nitric oxide in arginine-depleted cells leading to peroxynitrite-mediated cellular injury. Proc Natl Acad Sci U S A 93: 6770–6774. 8692893 10.1073/pnas.93.13.6770 PMC39102

[65] Pou S, Pou WS, Bredt DS, Snyder SH, Rosen GM (1992) Generation of superoxide by purified brain nitric oxide synthase. J Biol Chem 267: 24173–24176. 1280257

[66] Cardounel AJ, Cui H, Samouilov A, Johnson W, Kearns P, Tsai AL, et al (2007) Evidence for the pathophysiological role of endogenous methylarginines in regulation of endothelial NO production and vascular function. J Biol Chem 282: 879–887. 17082183 10.1074/jbc.M603606200

[67] Worrall NK, Chang K, LeJeune WS, Misko TP, Sullivan PM, Ferguson TB Jr., et al (1997) TNF-alpha causes reversible in vivo systemic vascular barrier dysfunction via NO-dependent and-independent mechanisms. Am J Physiol 273: H2565–2574. 9435588 10.1152/ajpheart.1997.273.6.H2565

[68] Leiper J, Murray-Rust J, McDonald N, Vallance P (2002) S-nitrosylation of dimethylarginine dimethylaminohydrolase regulates enzyme activity: further interactions between nitric oxide synthase and dimethylarginine dimethylaminohydrolase. Proc Natl Acad Sci U S A 99: 13527–13532. 12370443 10.1073/pnas.212269799 PMC129707

[69] Komarova Y, Malik AB (2010) Regulation of endothelial permeability via paracellular and transcellular transport pathways. Annu Rev Physiol 72: 463–493. 10.1146/annurev-physiol-021909-135833 20148685

[70] Wojciak-Stothard B, Torondel B, Tsang LY, Fleming I, Fisslthaler B, Leiper JM, et al (2007) The ADMA/DDAH pathway is a critical regulator of endothelial cell motility. J Cell Sci 120: 929–942. 17327280 10.1242/jcs.002212

[71] Wojciak-Stothard B, Torondel B, Zhao L, Renne T, Leiper JM (2009) Modulation of Rac1 activity by ADMA/DDAH regulates pulmonary endothelial barrier function. Mol Biol Cell 20: 33–42. 10.1091/mbc.E08-04-0395 18923147 PMC2613095

[72] Qin L, Qin S, Zhang Y, Zhang C, Ma H, Li N, et al (2014) p120 Modulates LPS-Induced NF- kappa B Activation Partially through RhoA in Bronchial Epithelial Cells. Biomed Res Int 2014: 932340 10.1155/2014/932340 24995336 PMC4065672

[73] Perona R, Montaner S, Saniger L, Sanchez-Perez I, Bravo R, Lacal JC (1997) Activation of the nuclear factor-kappaB by Rho, CDC42, and Rac-1 proteins. Genes Dev 11: 463–475. 9042860 10.1101/gad.11.4.463

[74] Yakovlev VA, Barani IJ, Rabender CS, Black SM, Leach JK, Graves PR, et al (2007) Tyrosine nitration of IkappaBalpha: a novel mechanism for NF-kappaB activation. Biochemistry 46: 11671–11683. 17910475 10.1021/bi701107z PMC2678910

